# Mental Health and Well-Being in Racial or Ethnic Minority Individuals After Using a Faith and Prayer Mobile App (Pray.com): Feasibility and Preliminary Efficacy Trial

**DOI:** 10.2196/52560

**Published:** 2024-02-02

**Authors:** Breanne Laird, Sabrina Zuniga, Joshua N Hook, Daryl R Van Tongeren, Lynda Joeman, Jennifer Huberty

**Affiliations:** 1 Pray, Inc Westlack Village, CA United States; 2 Department of Psychology University of North Texas Denton, TX United States; 3 Department of Psychology Hope College Holland, MI United States; 4 Tombridge United Kingdom; 5 Fit Minded, Inc Phoenix, AZ United States

**Keywords:** religion, spirituality, mobile apps, mental health, well-being, app, ethnic, technology, engagement, stress, depression, anxiety, quality of life, spiritual well-being, racial, spiritual practices, spiritual practice, mobile phone, mobile health, mHealth

## Abstract

**Background:**

Research is needed on how faith and prayer apps fit within the values of racial and ethnic minority (REM) groups, as well as whether such apps are effective in promoting mental health and well-being.

**Objective:**

This study aims to determine the feasibility and preliminary effectiveness of using the mobile app Pray.com on mental health and well-being among REM participants.

**Methods:**

This study was a single-group (N=77), 4-week feasibility trial in REM groups (65/77, 84% Black or African American). Participants were asked to use the Pray.com app at no cost for at least 5 times per week for 5 minutes per day. Participants completed questionnaires at the baseline and postintervention time points. Feasibility questionnaires were only completed at the postintervention time point, including qualitative interviews (n=15). The feasibility questions included acceptability (ie, satisfaction, intent to continue use, perceived appropriateness, and fit within culture), demand (ie, self-reported app use, expressed interest, and perceived demand), and practicality (ie, ease or difficulty of use, ability to use the app, and cost-effectiveness). Frequency and descriptive statistics were used to analyze feasibility outcomes. Changes in dependent variables were analyzed using paired-sample 2-tailed *t* tests. Partial correlations were conducted to explore the association between app use and outcomes, controlling for baseline scores.

**Results:**

Participants reported (54/72, 75% responded with “very likely” or “likely” to the feasibility questions) that they perceived the Pray.com app as acceptable. These findings were supported by qualitative interviews (n=15). Most participants (62/72, 86%) did not meet the app use prescription but expressed interest in using the app in the future and perceived demand for it in their communities. In addition, participants reported that the app was easy to use and perceived it to be inexpensive (US $7.99). Participants reported improved mental health (ie, stress and depressive and anxiety symptoms) and well-being (ie, satisfaction with life, spiritual well-being, religious commitment, and racial or ethnic identity development) at postintervention despite relatively low average levels and high variability of app use (average total of 45.83, SD 111.90 min over the course of the study). Greater app use was significantly associated with improvements in mental health and spiritual well-being. However, app use and study methodology limitations suggest that the study results may not accurately capture the full impact of Pray.com use.

**Conclusions:**

This is the first study to assess the feasibility of a faith and prayer app for mental health and well-being in a sample of REM individuals. Our findings suggest that the use of a faith and prayer app (ie, Pray.com) could be feasible and significantly impactful for the improvement of mental health symptoms and well-being in REM individuals and their communities, especially Black and African American individuals with a Christian affiliation. Further research is warranted.

## Introduction

### Background

Religious and spiritual identity can extend across and within various cultures, even worldwide, although religious experiences are largely influenced by and intersect with cultural context. Some have conceptualized religion as a form of culture [1], and previous research has examined how religious expressions differ by culture [2]. Religion can serve as a primary social identity [3] that intersects with other salient features of identity, such as one’s racial or ethnic identity [4]. Put simply, one’s religious experience and expression notably intersect with one’s cultural identity and context.

Despite the heterogeneity of religious expressions, researchers have identified certain core dimensions of religion, including cognitive, emotional, behavioral, and social features [5]. A substantial body of research suggests that religion enhances individual well-being [6-16]. However, less is known about the role of culture in the connection between religion and individual health and well-being.

For racial and ethnic minority (REM) populations, the religious or spiritual aspect of one’s identity might be particularly important. For example, Constantine and Sue [17] outlined a theoretical model describing optimal human functioning for people of color in the United States. One of the key premises of their argument was that one’s cultural context affects what is considered optimal human functioning for various groups and it is necessary to consider the impact of cultural values on the well-being of people of color. In their model, cultural values, beliefs, and practices such as collectivism, racial and ethnic pride, spirituality and religion, interconnectedness of mind, body, or spirit, and family and community are viewed as important predictors of optimal human functioning for people of color.

In this study, we focused on religion or spirituality as an important factor contributing to the mental health and well-being of REM groups. Indeed, research has supported the importance of religion in the lives of such groups (eg, African American and Latinx groups), and these individuals tend to engage in religious or spiritual practices at a higher rate than the US population overall [18,19]. For example, studies have found high levels of religious participation in African American individuals (ie, 89% are religious; 78% attend services regularly; and 90% pray, meditate, or use religious materials) [20]. Furthermore, previous research has supported a strong connection between religiousness and well-being in African American individuals [21,22]. For example, in a sample of African American individuals, racial or ethnic identity was positively related to both satisfaction with and meaning in life, and these relationships were partially mediated by religious commitment [23].

Similarly, engaging in religious practices has been linked to increased social support and psychological well-being and decreased perceived stress and physical pain in Latinx populations [24,25]. Furthermore, types of religious coping (eg, positive vs negative) affected symptoms of both depression and obsessive-compulsive disorder [24,26]. As religiosity tends to be more emphasized by Latinx immigrants in comparison with US-born Latinx populations [27-29], some scholars have hypothesized that a decline in religiosity may partially explain the Latino health paradox, in which more recently immigrated Latinx individuals tend to display better health outcomes than more assimilated and US-born Latinx individuals [30,31].

Regarding Asian and Asian American populations, religious identity was positively associated with self-esteem, positive affect, and meaning in life in a sample of Asian American adolescents [32]. In a sample of Chinese American and Korean American older adults, religious coping and support were associated with increased life satisfaction and decreased depressive symptoms [33]. In a sample of Korean adult immigrants, religious support was associated with higher well-being in the midst of experiencing financial hardship and difficulties with English [34]. Interviews with Filipino American individuals regarding their faith revealed that their experiences with religion and spirituality had both positive and negative effects on their well-being [35], although recent research has proposed that the negative effects of religion may be explained by scrupulosity in communities in which religious participation plays a large role in culture [26].

This high degree of religious participation by REM individuals is observed not only in the United States but also worldwide. For example, high rates of religious identification are observed in territories with high numbers of REM individuals, such as Latin America or the Caribbean, Middle East or North Africa, and sub-Saharan Africa [36]. Overall, it is clear that, for various REM populations, religious and spiritual identity are important factors that affect one’s quality of life. Thus, it is imperative to identify how religious identity, practices, and engagement empower REMs and assess how current practices can be implemented, changed, or improved to enhance religious and spiritual experiences and increase well-being in these populations.

### Different Pathways to Religious and Spiritual Well-Being

Technological advances have permitted various ways for individuals to facilitate well-being through engagement in religious and spiritual practice [37-39]. Although various media have previously encouraged several methods toward transcendent connection, the global health pandemic of 2020 accelerated the development of digital tools that allow individuals to enhance their well-being via religious and spiritual practices [37,38]. Many of these behavior changes have persisted, and individuals are engaging in technologically mediated religious and spiritual practices more regularly.

For example, individuals may interact with religious and spiritual content through mobile apps. One such app is Pray.com, which is an app that allows participants to engage in Christian-based faith and prayer content, such as reading the Bible, listening to sermons, or hearing stories or other creative content to engage with their spiritual faith. This app can be downloaded to any smartphone or internet-enabled (mobile) device, and it includes the opportunity to receive notifications to remain engaged in one’s religious practices. The Pray.com app was chosen for this study because of its popularity and potential reach but was not specifically designed for REM individuals.

Such research on the role of technology in religious and spiritual practices is nascent, and most research has been conducted on the Christian faith as it is a prominent part of public and political life in the United States [40]. Among the largest religious groups in the United States, Black Protestant denominations tend to use technology in their religious practices to a greater degree [41], yet digitally based interventions struggle to be representative of REM groups [42]. Thus, there is a dearth of research including REM diverse samples, and less research has focused on how such apps align with the cultural values of such participants. There is a need for research that explores how such apps fit (or do not fit) with the cultural values of REM participants as well as whether such apps are effective in promoting mental health and well-being. To our knowledge, this is the first study to assess the feasibility and preliminary efficacy of a faith and prayer app for mental health and well-being in REM individuals. Accordingly, the goal of this research was to examine how individuals self-identifying as belonging to REM groups engage with technologically mediated religious and spiritual practices to enhance their mental health and well-being.

### Study Overview and Hypothesis

The purpose of this study was to determine the feasibility (ie, acceptability, demand, and practicality) and preliminary effectiveness of using the mobile app Pray.com on mental health (ie, stress and depressive and anxiety symptoms) and well-being (ie, satisfaction with life, spiritual well-being, religious commitment, and racial or ethnic identity development) among REM participants. We predicted that Pray.com would be feasible in REM individuals and be associated with improved mental health and well-being outcomes at 4 weeks.

## Methods

### Ethical Considerations

This study was approved by the institutional review board of Biola University (F22-013) and registered at ClinicalTrials.gov (NCT05626673). All participants provided electronic consent before taking part.

### Study Design or Recruitment

This study was a single-group, 4-week feasibility trial. This method was chosen as this was a preliminary study of the feasibility and effectiveness of the app in a sample of REM individuals. Participants were recruited using convenience sampling through faith-based organizations (eg, churches and religious psychology organizations), personal contacts of the researchers, and social media between January 2023 and April 2023 and were directed to a link to complete the web-based eligibility survey.

### Participants and Procedure

Potential participants completed a web-based eligibility questionnaire via Qualtrics (Qualtrics International, Inc). Potential participants were eligible if they (1) self-identified as belonging to an REM group, (2) were aged ≥18 years, (3) owned a smartphone and were willing to download a mobile app, and (4) were willing to engage in Christian-based religious practice on a mobile app. Eligible participants were directed to a web-based informed consent form and informed of the potential risks of taking part in the study. After signing the consent form, participants were asked to complete a series of web-based baseline questionnaires (time 1) related to mental health and well-being. Once the questionnaires were completed, participants were provided with instructions on how to download the Pray.com mobile app.

### Intervention

Participants were asked to use the Pray.com app at no cost for at least 5 times per week for 5 minutes per day. The Pray.com app was chosen because of its popularity and potential reach. The app was not specifically designed for REM individuals. This dose was chosen as frequent private prayer has been associated with significant mental health benefits [43], and frequency may be more important than the duration of time spent in prayerlike practices. We chose a brief minimum period to increase adherence and consistency. Participants were contacted at time 2 (approximately 30 days after completing time 1) and invited to complete the web-based questionnaires again as well as a feasibility questionnaire about their experience using the app. After completing the time 2 questionnaires, participants were compensated with a digital gift card, debriefed about the purpose of the study, and invited to contact the researchers if they had any questions about their participation. A subset of participants (n=15) was invited (using random selection) to participate in a qualitative interview with a member of the research team (making this a mixed methods study). These qualitative interviews were conducted on the web via Zoom (Zoom Video Communications) and transcribed (and subsequently coded by the qualitative data analyst). The participants who completed the qualitative interviews were compensated with an additional digital gift card.

### Measures

#### Feasibility (Time 2)

All feasibility measures were assessed at the postintervention time point (4 weeks). We measured feasibility using the guidelines by Bowen et al [44], including acceptability, demand, practicality, and preliminary efficacy (ie, trends in changes in stress, depressive and anxiety symptoms, satisfaction with life, spiritual well-being, religious commitment, and racial or ethnic identity development). Benchmarks for feasibility included (1) acceptability (ie, satisfaction, intent to continue use, perceived appropriateness, and fit within culture; ≥75% of participants reporting satisfaction with the app and that the app was appropriate and fit within their cultural identity or worldview), (2) demand (ie, self-reported app use, expressed interest, and perceived demand; ≥75% of participants adhering to the Pray.com prescription, expressing interest in future use, and perceiving the app as demanded within their community), and (3) practicality (ie, ease or difficulty of use, ability to use the app, and cost-effectiveness; ≥75% of participants reporting ease of use and that they could pay for the app).

The feasibility measures were developed by psychology and behavioral health researchers. Textbox 1 presents a list of the feasibility questions.

A subset of participants (n=15) completed a semistructured qualitative interview that focused on exploring their experiences using the app in a deeper way. The qualitative interview focused on topics similar to those in the feasibility questionnaire (eg, acceptability, demand, and practicality), as well as including an open-ended question asking for general feedback about the app. Textbox 2 presents a list of the interview questions.

**Feasibility questions.**

**Acceptability**

- Satisfaction
  How satisfied were you with the Pray.com app?
- Intent to continue use
  How likely are you to continue to use Pray.com?
- Perceived appropriateness
  How relevant was the Pray.com app to improving your mental health?
  My racial/ethnic identity is well represented on the Pray.com app?
  The material on the Pray.com app was a good fit with my racial/ethnic identity?
  The material on the Pray.com app was a good fit with my cultural worldview?
- Fit within culture
  How well did using the Pray.com app fit within your religious beliefs or worldview?
  How well did using the Pray.com app fit within your cultural (ie, racial or ethnic) identity or worldview?

**Demand**

- Self-reported use
  How often did you use the Pray.com app?
- Expressed interest
  How interested are you in using the Pray.com app in the future?
- Perceived demand
  How much demand do you think there would be for the Pray.com app within your religious community?
  How much demand do you think there would be for the Pray.com app within your culture?

**Practicality**

- Ease or difficulty of use
  How easy or difficult was it to use the Pray.com app?
- Ability to use the app
  How would you rate your ability to use and navigate Pray.com?
- Cost
  How cheap or expensive does this price seem to you?
  How easy or difficult would it be to pay the monthly fee for the Pray.com app?
  How likely would you be willing to pay the monthly fee for the Pray.com app?

**Interview questions.**

**Acceptability**

- Satisfaction
  What did you like the most about using the Pray.com app?
  What did you like least about using the Pray.com app?
- Intent to continue use
  What factors would influence your decision to continue using the Pray.com app in the future?
  What factors would keep you from using the Pray.com app after the study is over?
- Perceived appropriateness
  What about the Pray.com app was most relevant to helping you with your mental health?
  What about the Pray.com app was most relevant to helping your spiritual life?
  How did Pray.com contribute to how you feel about meditation/mindfulness?
- Fit within culture
  What aspects of using the Pray.com app were a good fit within your religious beliefs or worldview?
  What aspects of using the Pray.com app did not seem to fit within your religious beliefs or worldview?
  What aspects of using the Pray.com app were a good fit within your cultural (ie, racial/ethnic) identity or worldview?
  What aspects of using the Pray.com app did not seem to fit within your cultural (ie, racial/ethnic) identity or worldview?
  How well represented was your racial/ethnic group on the Pray.com app?

**Demand**

- Expressed interest
  What aspects of the Pray.com app are you most interested in?
  What aspects of the Pray.com app are you least interested in?
- Perceived demand
  What could be improved about the Pray.com app to make it a better fit for you?
  What could be improved about the Pray.com app to make it a better fit for your religious community?

**Practicality**

- Ease or difficulty of use
  What aspects of the Pray.com app were difficult to use?
  What aspects of the Pray.com app were easy to use?
- Ability to use the app
  What aspects of the Pray.com app did you feel like you had the ability to use?
  Was there anything about the Pray.com app that you felt like you did not have the ability to use?
- Other
  Do you have any other feedback about your use of the Pray.com app?

#### Perceived Stress Scale (Time 1 and 2)

The Perceived Stress Scale-10 (PSS-10) [45] was used to measure participants’ subjective levels of stress. The PSS-10 is a 10-item measure in which participants rate items related to stress (eg, “how often have you been angered because of things that happened that were outside of your control?”) on a scale from 0=*never* to 4=*very often* within the previous month. A mean perceived stress score was calculated, with higher scores indicating higher levels of perceived stress. Previous research has demonstrated high reliability and construct validity of the PSS-10 [45,46], including in a sample of REM participants [47]. In this sample, the Cronbach α for the PSS-10 ranged from .81 to .84 across time points.

#### Hospital Anxiety and Depression Scale (Time 1 and 2)

The Hospital Anxiety and Depression Scale (HADS) [48] was used to measure participants’ symptoms of anxiety and depression. The HADS is a 14-item measure in which participants rate 7 items related to anxiety (eg, “I feel tense or ‘wound up.’”) and 7 items related to depression (eg, “I still enjoy the things I used to enjoy” [reverse coded]) on a scale from 0 to 3 (end points vary across items). Mean scores for anxiety and depression were calculated, with higher scores indicating more severe symptoms of anxiety and depression. Previous research has demonstrated evidence for the reliability and construct validity of the HADS [49], including cross-culturally [50]. In this study, the Cronbach α for the anxiety and depression subscales of the HADS ranged from .83 to .83 (anxiety) and from .79 to .84 (depression) across time points.

#### Spiritual Well-Being Scale (Time 1 and 2)

Participants’ spiritual well-being was assessed using the Spiritual Well-Being Scale (SWBS) [51,52]. The SWBS comprises 20 items divided into 2 subscales: religious well-being (10 items; eg, “I feel most fulfilled when I’m in close communion with God”) and existential well-being (10 items; eg, “I believe there is some real purpose for my life”). Participants rated each item on a scale from 1=*strongly disagree* to 6=*strongly agree*, with higher scores indicating higher levels of spiritual well-being. The SWBS has previously demonstrated high internal consistency and construct validity [51,52], including in samples with African American individuals [53]. In this study, the Cronbach α for the SWBS ranged from .91 to .92 across time points.

#### Satisfaction With Life Scale (Time 1 and 2)

Participants’ life satisfaction was measured using the Satisfaction With Life Scale (SWLS) [54]. Participants rated 5 items related to life satisfaction (eg, “In most ways my life is close to my ideal”) on a scale from 1=*strongly disagree* to 6=*strongly agree,* with higher scores suggesting higher life satisfaction. The SWLS has demonstrated high internal consistency and construct validity in previous research across various populations and contexts [54], including diverse populations [55]. In this study, the Cronbach α for the SWLS ranged from .73 to .83 across time points.

#### Religious Commitment Inventory–10 (Time 1 and 2)

Religious commitment was measured using the Religious Commitment Inventory-10 (RCI-10) [56]. Participants rated 10 items assessing religious commitment (eg, “I spend time trying to grow in understanding of my faith”) on a scale from 1=*not true at all of me* to 5=*totally true of me*. Scores were measured using the mean of all 10 items, with higher scores suggesting higher levels of religious commitment. The RCI-10 has shown evidence of internal consistency and construct validity [56], including in diverse samples [56,57]. In this study, the Cronbach α for the RCI-10 ranged from .84 to .88 across time points.

#### Multigroup Ethnic Identity Measure–Revised (Time 1 and 2)

Racial or ethnic identity development was measured using the Multigroup Ethnic Identity Measure–Revised (MEIM-R) [58]. Participants rated 6 items assessing racial or ethnic identity development across 2 subscales—commitment (eg, “I have a strong sense of belonging to my own ethnic group”) and exploration (eg, “I have spent time trying to find out more about my ethnic group, such as its history, traditions, and customs”)—on a scale from 1=*strongly disagree* to 5=*strongly agree*, with higher scores indicating higher levels of commitment and exploration, respectively. The MEIM-R has demonstrated evidence of internal consistency and construct validity [58]. In this study, the Cronbach α for the MEIM-R ranged from .79 to .79 across time points.

### Statistical Analysis

The data were analyzed using SPSS (version 28.0; IBM Corp). Frequency and descriptive statistics were used to analyze the feasibility outcomes and demographic characteristics. As not all participants answered every question (ie, it was a free-response survey), the sample sizes differed across analyses. We reported all available completed data. Self-reported use frequency was categorized as ordinal, reflecting the use of the app as (1) less than once per week, (2) up to 2 times per week, (3) 3 to 5 times per week, or (4) ≥5 times per week. All other feasibility questions were categorized as ordinal. Changes in dependent variables between time 1 and time 2 were analyzed using paired-sample 2-tailed *t* tests. To assess the relationship between objective app use and changes in dependent variables, partial correlations were conducted to explore the association between app use and scores on time 2 variables controlling for scores on time 1 variables. *P* values of <.05 were considered significant.

With regard to the qualitative data, interview transcripts were imported into NVivo (QSR International) for coding and analysis. Thematic analysis methods were used based on the inductive or deductive approach proposed by Braun and Clarke [59]. In brief, this involved first identifying top-level themes based on the main issues and questions covered in the interviews (feasibility and mental health and well-being outcomes). Within these, emergent themes were identified inductively from the transcripts and labeled appropriately. An iterative process was used in which relevant extracts of interview data were allocated to these, with the coding being continually reviewed and revised until the analyst felt confident that it most accurately reflected the lived experiences of the research participants as reported in their interviews.

## Results

Of the 77 final participants in our study, 72 (94%) completed time 2 measures. There were no significant differences in our main variables between completers and noncompleters.

### Participants

We received 707 initial responses to the eligibility survey; however, the vast majority of these were found to be inauthentic responses (eg, bots and duplicate IP addresses). Accordingly, we checked the data using elimination of duplicate IP addresses and consistent responses to demographic information. Then, we installed various data quality checks such as bot detection and reCAPTCHA (Google) and accuracy on 3 quality-check questions (eg, “Please select disagree”). This resulted in a final sample of 77 participants. Table 1 shows the sample demographic characteristics. Table 2 shows the religious or spiritual engagement at the beginning of the study.

**Table 1:** Sample demographics (N=77).

Demographics		Values
Age (y), mean (SD)		31 (5)
**Gender, n (%)**		
	Cisgender women	23 (30)
	Cisgender men	54 (70)
**Race or ethnicity, n (%)**		
	American Indian or Alaska Native	6 (8)
	Asian or Asian American	1 (1)
	Black or African American	65 (84)
	Latina, Latino, or Hispanic	3 (4)
	Native Hawaiian or Pacific Islander	2 (3)
**Sexual orientation, n (%)**		
	Heterosexual	74 (96)
	Gay	1 (1)
	Lesbian	1 (1)
	Bisexual	1 (1)
**Religious affiliation, n (%)**		
	Christian (Catholic)	42 (55)
	Christian (evangelical Protestant)	19 (25)
	Christian (mainline Protestant)	4 (5)
	Christian (Black Protestant)	5 (6)
	Buddhist	1 (1)
	Hindu	1 (1)
	Jewish	1 (1)
	None	1 (1)
**Religious identity, n (%)**		
	Stably religious	56 (73)
	New identifier	8 (10)
	Reidentifier	12 (16)
	Never identified	1 (1)

**Table 2:** Religious or spiritual engagement at the beginning of the study (N=77).

Engagement characteristics			Participants, n (%)
**Religious service attendance**			
	At least once a week	57 (74)	
	Once or twice a month	18 (23)	
	Seldom or never	2 (3)	
**Prayer frequency**			
	At least once a week	54 (70)	
	Once or twice a month	19 (25)	
	Seldom or never	4 (5)	
**Participation in prayer, scripture, or religious education groups**			
	At least once a week	43 (56)	
	Once or twice a month	29 (38)	
	Seldom or never	4 (5)	
**Meditation frequency**			
	At least once a week	43 (56)	
	Once or twice a month	26 (34)	
	Seldom or never	8 (10)	
**Scripture reading**			
	At least once a week	47 (61)	
	Once or twice a month	21 (27)	
	Seldom or never	8 (10)	

### Feasibility

#### Acceptability

All the acceptability benchmarks were met. Most participants (64/72, 89%) reported being very satisfied or satisfied with the app and that they were very likely or likely to continue to use the app in the future (63/72, 88%). No significant differences between men and women were observed regarding app acceptability.

The app was perceived as appropriate. Most participants reported that using the app was very relevant or relevant to improving their mental health (57/72, 79%) and spiritual lives (62/72, 86%). Most participants (60/72, 83%) reported that they strongly agreed or agreed that their racial or ethnic identity was well represented on the app and that the content was a good fit with their racial or ethnic identity (53/72, 74%) and cultural worldview (59/72, 82%). Most participants reported that they strongly agreed or agreed that Pray.com was a good fit with their religious beliefs or worldview (58/72, 81%) and cultural identity or worldview (55/72, 76%).

Participants reported that the app was more relevant to improving their spiritual life than their mental health (*P*=.01). There was no significant difference between fit with religious or cultural worldview (*P*=.23). Table 3 shows participant ratings of the acceptability of the app.

**Table 3:** Participant ratings of the acceptability of the app (N=72).

Acceptability characteristics				Values
**Satisfaction, mean (SD)**				4.50 (0.69)
	Very satisfied, n (%)		44 (61)	
	Satisfied, n (%)		20 (28)	
	Somewhat satisfied, n (%)		8 (11)	
	Dissatisfied, n (%)		0 (0)	
**Intent to continue use, mean (SD)**				4.47 (0.71)
	Very likely, n (%)		43 (60)	
	Likely, n (%)		20 (28)	
	Somewhat likely, n (%)		9 (12)	
	Not likely, n (%)		0 (0)	
**Perceived appropriateness**				
	**Relevance in improving mental health, mean (SD)**		4.14 (0.74)	
		Very relevant, n (%)	25 (35)	
		Relevant, n (%)	32 (44)	
		Somewhat relevant, n (%)	15 (21)	
		Irrelevant, n (%)	0 (0)	
		Very irrelevant, n (%)	0 (0)	
	**Relevance in improving spiritual life, mean (SD)**		4.42 (0.88)	
		Very relevant, n (%)	44 (61)	
		Relevant, n (%)	18 (25)	
		Somewhat relevant, n (%)	7 (10)	
		Irrelevant, n (%)	2 (3)	
		Very irrelevant, n (%)	1 (1)	
**Fit within culture**				
	**Racial or ethnic identity well represented, mean (SD)**		4.29 (0.74)	
		Strongly agree, n (%)	33 (46)	
		Agree, n (%)	27 (38)	
		Somewhat agree, n (%)	12 (17)	
		Disagree, n (%)	0 (0)	
	**Content good fit with racial or ethnic identity, mean (SD)**		4.15 (0.85)	
		Strongly agree, n (%)	31 (43)	
		Agree, n (%)	22 (31)	
		Somewhat agree, n (%)	18 (25)	
		Disagree, n (%)	1 (1)	
	**Content good fit with cultural worldview, mean (SD)**		4.29 (0.76)	
		Strongly agree, n (%)	34 (47)	
		Agree, n (%)	25 (35)	
		Somewhat agree, n (%)	13 (18)	
		Disagree, n (%)	0 (0)	
	**Content good fit with religious beliefs or worldview, mean (SD)**		4.25 (0.87)	
		Very good fit, n (%)	35 (49)	
		Good fit, n (%)	23 (32)	
		Somewhat good fit, n (%)	11 (15)	
		Poor fit, n (%)	3 (4)	
	**Fit with cultural identity or worldview, mean (SD)**		4.11 (0.87)	
		Very good fit, n (%)	28 (39)	
		Good fit, n (%)	27 (38)	
		Somewhat good fit, n (%)	14 (19)	
		Poor fit, n (%)	3 (4)	

#### Demand

All demand benchmarks were met except for self-reported use (at least 75% of participants used the app at least 5 times per week for 5 min/d) as this could not be determined from the answer choices selected by the participants. Most participants (58/72, 81%) self-reported that they used the app between 3 and 5 times per week (35/72, 49%) or >5 times per week (23/72, 32%). However, objective use data showed that participants used the app for an average of 45.83 (SD 111.90) minutes and a total of 5.77 (SD 8.19) days throughout the entire study period, suggesting that app use may have been overreported. Regardless of app use, most participants (61/72, 85%) indicated that they were extremely interested or interested in continuing to use the app in the future and that they believed that there would be interest or a great deal of interest in the app in both their religious (59/72, 82%) and cultural communities (56/72, 78%). There was no significant difference between perceived demand in one’s religious community and in one’s cultural community (*P*=.13). No significant differences were observed between men and women regarding demand for the app. Table 4 shows participant ratings regarding demand for the app.

**Table 4:** Participant ratings regarding demand for the app (N=72).

Demand characteristics				Values
**Frequency of use, mean (SD)**				3.11 (0.74)
	>5 times/wk, n (%)		23 (32)	
	3-5 times/wk, n (%)		35 (49)	
	1-2 times/wk, n (%)		13 (18)	
	Less than once/wk, n (%)		1 (1)	
**Interest in future use, mean (SD)**				4.31 (0.73)
	Extremely interested, n (%)		33 (46)	
	Interested, n (%)		28 (39)	
	Somewhat interested, n (%)		11 (15)	
	Not interested, n (%)		0 (0)	
**Perceived demand**				
	**Within participants’ religious community, mean (SD)**		4.29 (0.83)	
		A great deal, n (%)	36 (50)	
		Interested, n (%)	23 (32)	
		Somewhat interested, n (%)	11 (15)	
		Not really, n (%)	2 (3)	
	**Within participants’ cultural community, mean (SD)**		4.11 (0.82)	
		A great deal, n (%)	26 (36)	
		Interested, n (%)	30 (42)	
		Somewhat interested, n (%)	14 (19)	
		Not really, n (%)	2 (3)	

#### Practicality

Almost all participants (58/72, 81%) reported that it was very easy or easy to use the app and that they were very able or able to use and navigate the app (63/72, 88%). The monthly price of the app was perceived to be very cheap or cheap by most participants (55/72, 76%). Although only half (39/72, 54%) reported that paying the monthly fee of the app would be very easy or easy, most participants (56/72, 78%) reported that they would be very likely or likely to pay the monthly fee. No significant differences were observed between men and women regarding the practicality of the app. Table 5 shows participant ratings regarding the practicality of the app.

**Table 5:** Participant ratings regarding the practicality of the app (N=72).

Practicality characteristics			Values
**Difficulty of use, mean (SD)**			1.76 (0.90)
	Very easy, n (%)	35 (49)	
	Easy, n (%)	23 (32)	
	Somewhat easy, n (%)	10 (14)	
	Difficult, n (%)	4 (6)	
**Ability to use and navigate the app, mean (SD)**			4.35 (0.70)
	Very high ability, n (%)	34 (47)	
	Able, n (%)	29 (40)	
	Somewhat able, n (%)	9 (12)	
	Not able, n (%)	0 (0)	
**Perceived cost of the app, mean (SD)**			2.46 (0.86)
	Very cheap, n (%)	29 (40)	
	Cheap, n (%)	26 (36)	
	Somewhat cheap, n (%)	8 (11)	
	Expensive, n (%)	0 (0)	
**Ease of paying monthly fee, mean (SD)**			2.35 (0.97)
	Very easy, n (%)	16 (22)	
	Easy, n (%)	23 (32)	
	Somewhat easy, n (%)	26 (36)	
	Difficult, n (%)	6 (8)	
	Very difficult, n (%)	1 (1)	
**Willingness to pay monthly fee, mean (SD)**			4.14 (0.83)
	Very likely, n (%)	28 (39)	
	Likely, n (%)	28 (39)	
	Somewhat likely, n (%)	14 (19)	
	Unlikely, n (%)	2 (3)	

### Mental Health and Well-Being Improvement Over Time

Regarding mental health symptoms, participants reported significant improvement in perceived stress (*P*=.004; *d*=0.35), depression (*P*=.02; *d*=0.29), and anxiety (*P*=.01; *d*=0.32). Effect sizes were in the small to medium range. Regarding well-being, participants reported significant improvement in satisfaction with life (*P*<.001; *d*=0.52), spiritual well-being (*P*<.001; *d*=0.48), religious commitment (*P*=.02; *d*=0.28), and racial or ethnic identity development (*P*=.048; *d*=0.24). Effect sizes were in the small to medium range. Table 6 shows the *t* test reporting of improvements.

**Table 6:** Paired-sample 1-tailed *t* test for mental health and well-being outcome improvement from time 1 and time 2.

Variable	Time 1, mean (SD)	Time 2, mean (SD)	*t* test (*df*)	*P* value	Cohen *d* (effect size)
Stress	2.72 (0.66)	2.52 (0.71)	2.95 (76)	.004	0.35
Depression	0.84 (0.53)	0.70 (0.57)	2.46 (76)	.02	0.29
Anxiety	1.12 (0.59)	0.98 (0.58)	2.71 (76)	.01	0.32
Religious commitment	3.84 (0.71)	4.01 (0.61)	−2.35 (76)	.02	0.28
Spiritual well-being	4.47 (0.80)	4.89 (0.83)	−4.10 (76)	<.001	0.48
Satisfaction with life	4.68 (1.26)	5.31 (0.97)	−4.43 (76)	<.001	0.52
Racial or ethnic identity	3.96 (0.63)	4.09 (0.58)	−2.01 (76)	.048	0.24

### Mental Health and Well-Being Improvement With Objective App Use

Participants used the app during the study period (approximately 4 weeks) for an average of 45.83 (SD 111.90) minutes and a total of 5.77 (SD 8.19) days. Partial correlations were conducted, controlling for baseline variable scores, between total minutes and total days of app use and outcome variables. When controlling for the outcome variable at baseline, greater app use was associated with decreased stress and depressive and anxiety symptoms and increased spiritual well-being at the postintervention time point (Table 7). These results suggest that greater app use is associated with decreased mental health symptoms and increased spiritual well-being over time.

**Table 7:** Partial correlations between app use and dependent variables at the postintervention time point (N=77).

	Total minutes	*P* value	Total days	*P* value
Stress	−0.20	.10	−0.30	.01
Depression	−0.27	.02	−0.29	.01
Anxiety	−0.36	.002	−0.39	.001
Religious commitment	0.16	.20	0.22	.07
Spiritual well-being	0.26	.03	0.30	.01
Satisfaction with life	0.15	.22	0.20	.09
Racial or ethnic identity	0.12	.32	0.06	.67

### Qualitative Analyses

#### Acceptability

When the interview participants (n=15) were asked what they liked most about the app, the types of responses fell into 2 main categories: general aspects and specific features.

##### General Aspects of the App

In total, 20% (3/15) of the participants reported that they mostly liked the wide range of topics and content available on the app, whereas 13% (2/15) referred to its relatability. Others (3/15, 20%) mentioned liking the most how convenient the app was to use via their phone, compared with hard-copy methods of accessing spiritual content, and how educational it was for them (2/15, 13%). Other participants mentioned liking the community aspect of the app (1/15, 7%) and the overall look of the app and its interface (1/15, 7%).

##### Specific Features of the App

A total of 27% (4/15) of the interviewees reported that they most liked the notifications that reminded them to pray regularly, and 13% (2/15) reported that they particularly liked the podcasts: “I’ve been getting addicted to the podcasts very early in the morning.”

In total, 27% (4/15) of the interview participants mentioned that they especially liked the daily scriptures or Bible passages. Other favorite features mentioned by individual participants included the daily motivational messages (1/15, 7%), daily prayer time (1/15, 7%), and streaks (1/15, 7%) enabling them to track their own progress. The participants were also asked which aspects of the app they were most interested in. In descending order of frequency in which they were mentioned, these were reported to be daily prayers (6/15, 40% of the participants), preaching and sermons (5/15, 33%), daily motivations (3/15, 20%), scriptures (3/15, 20%), podcasts (3/15, 20%), books (1/15, 7%), and storylines (1/15, 7%).

##### What Participants Liked the Least About the App

When asked what they liked the least about the Pray.com app, 60% (9/15) of participants reported that there was nothing they did not like about the app.

Among the 40% (6/15) who did report on features that they disliked or liked less, several of these were related to technical issues encountered when using the app (eg, delays in reminders, delays in content updates, and inability to save activity when exiting).

Individuals also commented on morning prayers being too long (1/6, 17%) and sermons being too short (1/6, 17%) and disliking the 21-Day Prayer Journeys (1/6, 17%). When asked which aspects of Pray.com they were the least interested in, 40% (6/15) indicated that there were no aspects they were uninterested in. A total of 13% (2/15) said that they were the least interested in certain preachers or those they knew less about, and 7% (1/15) referred to content perceived as irrelevant to them, such as anxiety meditations. In addition, some participants said that they were least interested in movies (2/15, 13%), meditations (1/15, 7%), prayer journals (1/15, 7%), and prayers (1/15, 7%).

##### Intent to Continue Use

We asked the participants what factors would influence their decision to continue using the Pray.com app. In total, 60% (9/15) indicated that they would continue to use it because of the beneficial effect it had had on their spirituality, especially the notifications that reminded them to pray. Others stated that the convenience and ease of use of the Pray.com app were factors that influenced their intention to continue using it (7/15, 47%) and that range, quality of content, or continued access to content (3/15, 20%) would influence their continued use.

##### Perceived Appropriateness

Many participants mentioned that they had experienced positive impacts of the Pray.com app on their mental health. A total of 27% (4/15) reported that these benefits were due to a feeling of greater connectedness, either with God or with others who share their religious beliefs. In total, 20% (3/15) described how they had benefited from the ease of being able to locate mental health content on the app. A total of 20% (3/15) of the participants reported that their levels of stress or depression had decreased since they had started using the app. Some participants cited specific types of content that had helped their mental health: meditations (3/15, 20%), daily podcasts (2/15, 13%), scriptures (1/15, 7%), and songs (1/15, 7%).

The participants were also asked how the app had influenced their feelings about meditation and mindfulness. In response, 40% (6/15) indicated that they had an improved understanding or that using the app had changed their misconceptions about meditation and mindfulness. In total, 27% (4/15) of the participants described how the app had helped them enter a meditative state or how much they had enjoyed the meditations.

##### Impacts on Spirituality

When asked about the impact of the Pray.com app on their spirituality, 33% (5/15) of the participants indicated that it had helped them develop spiritual habits or increase their familiarity with the scriptures. A total of 27% (4/15) of the participants referred to having increased their frequency of praying since they had started using the app. Another 27% (4/15) of the participants explained how the app had given them a more spiritual focus or direction in their lives. In total, 13% (2/15) of the interviewees described ways in which the app had taught them how to pray more effectively. A total of 13% (2/15) highlighted ways in which they had gained new spiritual insights or understanding through using the Pray.com app.

##### Fit Within Culture

When asked whether the Pray.com app was a good fit with their religious beliefs or worldview, 20% (3/15) of the participants commented that all aspects were a good fit. In total, 67% (10/15) of the participants specified ways in which particular content or preachers on the app were a good fit with their religious beliefs or worldview. When asked which aspects were not a good fit with their religious beliefs or worldview, 20% (3/15) cited specific content not relevant to them, such as parenting content, or that they or others in their community might not agree with, such as meditation or options for gift buying on the app.

##### Fit Within Cultural and Ethnic Identity

A total of 60% (9/15) of the interviewees specifically said that the Black community was well represented among the preachers. Although one participant expressed a perception that Black preachers only accounted for approximately 20% of all those on the app, he did not see this as a problem. A further 40% (6/15) of the interviewees similarly commented on how inclusive and nondiscriminatory Pray.com was. One participant mentioned that the app was a good fit with their cultural identity because of the type of language used, which he could relate to and understand.

#### Demand

In total, 80% (12/15) of the interviewees made suggestions for ways of improving the app to make it a better fit for them personally. These fell broadly into the following categories: features, technical improvements, and cost.

##### Features

Interviewees suggested more or different types of notifications, the ability to interact with others on the app (eg, in a forum or groups), or being able to post questions or comments to the preachers.

##### Technical Improvements

A total of 27% (4/15) of the participants said that technical improvements would make the app a better fit for them personally. These generally referred to specific technical problems they had encountered (eg, allowing the app to continue running while using the phone and inability to save content).

##### Cost

In total, 13% (2/15) of the participants suggested improvements related to the cost of subscriptions, including continued free access to the full app, a payment plan to make subscriptions more affordable, and a free trial option.

#### Practicality (Ease of Use)

Interviewees mentioned specific aspects or features of the app that were easy for them to use. A total of 40% (6/15) of the participants stressed that navigating the app was particularly easy for them. Other individuals commented that the podcasts or live prayers (3/15, 20%), meditations (1/15, 7%), website (1/15, 7%), and registration process (1/15, 7%) were easy to use. A small number of participants did describe some difficulties they had encountered when using the app. In total, 13% (2/15) of the participants explained that they had not realized that there was a notification option that they needed to activate. Others mentioned difficulty in locating the streak data (1/15, 7%) and using the display on the side of the podcasts (1/15, 7%).

## Discussion

### Principal Findings

#### Overview

The purpose of this study was to determine the feasibility (ie, acceptability, demand, and practicality) and preliminary effectiveness of using the mobile app Pray.com on mental health (ie, stress and depressive and anxiety symptoms) and well-being (ie, satisfaction with life, spiritual well-being, religious commitment, and racial or ethnic identity development) among REM participants. We also explored the associations between objective app use (total minutes and days) and the outcomes measured. In addition, follow-up interviews were conducted to provide further insight into feasibility outcomes. This is one of the first studies to assess the feasibility and preliminary effectiveness of a faith- and prayer-based app in REM individuals for the purpose of improving mental health and well-being.

Overall, participants reported high levels of satisfaction with the app, intent to continue use, appropriateness of the app, and fit within their culture. Interestingly, most participants (62/72, 86%) did not meet the app use prescription provided by the researchers (ie, at least 5 min/d, 5 d/wk) but expressed interest in using the app in the future and perceived demand for it in their communities. In addition, participants reported that the app was easy to use and perceived it to be inexpensive. Only approximately half (39/72, 54%) of the participants reported that paying the monthly fee would be easy for them, but most (56/72, 78%) said that they would be willing to pay. Even though most participants did not use the app as often as prescribed, the results indicated that app use was associated with significant mental health improvements over time. Critically, with regard to preliminary effectiveness, participants reported improved mental health (ie, stress and depressive and anxiety symptoms) and well-being (ie, satisfaction with life, spiritual well-being, religious commitment, and racial or ethnic identity development) over the course of the study despite relatively low average levels and high variability of app use. Greater app use was significantly associated with improvements in mental health and spiritual well-being. These findings suggest that Pray.com is feasible for REM adults. Future research is warranted to determine the efficacy of Pray.com on mental health and well-being outcomes in REM populations using randomized controlled trials.

#### Feasibility

Using a faith and prayer mobile app for mental health and well-being was found to be feasible in REM individuals, particularly for Black and African American adults (who constituted most of our sample). Most of the participants (64/72, 89%) expressed high satisfaction with the app and that the app was a good fit with their cultural and religious beliefs or worldview. A number of interviewees (6/15, 40%) also indicated that they perceived a fair representation of different cultural backgrounds in the app content. The findings related to cultural fit are promising as, historically, REM groups have not been well represented in apps, especially in apps that claim to have health benefits [60]. Ramos et al [61] have suggested that apps with a focus on improving mental health may have the ability to reduce unmet mental health needs if users believe that the content is appropriate for them and fits within their culture. Others have suggested that apps should be adapted to various cultures for increased benefits [42,62]. However, Black and African American populations are still underrepresented in mobile health research, limiting our ability to inform the development and design of apps for REM groups [63]. Our qualitative findings show that a small subset of participants reported that cultural fit could be increased if content such as parenting was improved or potential culturally controversial content such as meditation or options for gift buying on the app were addressed. This is one of the first studies to explore a faith-based app for mental health and well-being among REM groups. These initially promising findings suggest that more research in this area is warranted.

The findings on feasibility and cultural fit in our study are also exciting in light of the sometimes mixed reception of mindfulness and meditation in certain cultural and religious groups [64,65]. The positive reception and perceived cultural fit of the Pray.com app in a sample of religious REM individuals provides evidence that these apps may be an important avenue to help introduce the benefits of prayer, mindfulness, and meditation in a context that is experienced as a good fit with one’s cultural and religious worldview.

Although most participants (>54/72, 75%) reported high levels of feasibility across the board, one area in which responses were mixed was cost-effectiveness. Namely, although approximately three-quarters (56/72, 78%) of the participants expressed willingness to pay the monthly fee for the app, only approximately half (39/72, 54%) expressed that it would be easy for them to do so. This was supported by the qualitative data, in which some participants suggested improvements related to the cost of subscriptions (eg, a payment plan and free trial option). Individuals considering subscribing to an app for the purpose of improving or maintaining mental health and well-being should weigh the costs and potential benefits of subscribing to the app. Thus, when developing or implementing apps to provide access to faith and prayer content to various cultural groups, it is critical to keep cost-effectiveness and affordability in mind.

Previous research has demonstrated that overall product satisfaction and perceived quality are directly linked to increased purchase intentions, whereas increased product involvement may indirectly increase purchase intentions through overall satisfaction and perceived product quality [66,67]. Although only half of the sample expressed that it would be easy to pay the monthly fee, participants did say that paying the monthly fee was feasible and were interested in continuing use. Hence, despite some perceived expensiveness of the app, participants reported intent to continue use, likely based on high satisfaction with the app and its high relevance to improving mental and spiritual well-being.

#### Preliminary Effectiveness on Mental Health and Well-Being

Participants reported improved mental health as a result of using the Pray.com app. Specifically, participants reported significant improvements in mental health symptoms, including decreased stress, depression, and anxiety symptoms, over the course of the study. This was confirmed in the qualitative interviews; interviewees noted that their stress and depression levels decreased because of their increased hope and enjoyment of the present moment as encouraged by app content (eg, daily podcasts, scripture, and songs). In addition, the qualitative interviews suggested that it was easy to search for and access religious content related to stress, depression, and anxiety on the app. Prior work has demonstrated that spirituality can help operate as a buffer against stress and adversity [68]. To the degree that increased involvement with spiritual content can facilitate coping and help individuals in periods of stress, future research should explore potential mediators responsible for such benefits (eg, spiritual meaning [68]) and the contextual factors in which such benefits are maximized.

Interestingly, participants reported an initial hesitancy toward meditation, but interviewees reported that engagement with the meditation content on the app helped them enter a meditative state, which reportedly helped them face problems directly and better absorb information. This is an important finding given that some conservative Christians may hold negative views toward meditation because of its relationship with Buddhism [65]. For example, some more theologically conservative religious individuals may mistrust practices founded on traditions outside their own religion or view them as taboo. Accordingly, there might be hesitation to engage with content that does not squarely align with one’s religiously endorsed practices or that may not be prescribed by one’s religious teachings. Future work should explore the boundary conditions of these hesitations and what predicts such attitudes toward these practices.

Furthermore, the sample reported significantly greater well-being over time over the course of the study (ie, spiritual well-being, religious commitment, life satisfaction, and racial or ethnic identity development). Along these lines, interviewees expressed that engagement with the app (eg, podcasts, scripture reading, and meditations) positively affected their faith, spiritual well-being, and daily spiritual practices. Such findings are consistent with those of previous research suggesting that religious and spiritual identity and engagement are positively associated with well-being across REM groups [22,24,32]. Our findings are also consistent with those of other app-based mental health interventions that suggest that apps may serve as a self-management tool or adjunctive treatment [69,70]. However, app-based interventions have typically lacked diversity in their samples, and therefore, considering the preliminary nature of this study, outcome consistency cannot yet be determined [61].

The finding that participants reported higher levels of racial or ethnic identity over the course of the study was interesting and warrants further exploration. Religion and spirituality have been theorized to be important cultural factors in promoting positive psychological well-being in people of color [17]. For example, in a sample of African American individuals, religious commitment was related to higher levels of racial or ethnic identity and satisfaction with life [23]. It may be that the religious content on the app can provide a way to help encourage positive messages about one’s racial or ethnic identity and help people of color cope with and counter negative messages or narratives related to one’s racial/ethnic identity that are experienced in the broader culture (eg, racism and microaggressions). To be sure, future research sampling from a broader range of REM groups is important.

Given that the literature consistently demonstrates a significant association between religion or spirituality and well-being among REM populations, this app may provide a supplemental digital avenue to increase mental and spiritual well-being among REM groups, with the potential for greater improvement with increased use. This is especially important given the historical lack of access to mental health resources among African American and other REM individuals [71]. Future research should use rigorous methodological designs that rely on random assignment to experimental and control conditions, such as randomized clinical trials, to establish the causal effect of using a faith and prayer app for the purpose of improving mental health and well-being in REM groups.

#### Mental Health and Well-Being Improvement Association With Objective App Use

There was considerable variability in the degree to which participants used the app. Not all participants adhered to the instructions to use the app 5 minutes a day, 5 days per week. Greater objective app use was associated with decreased stress as well as depressive and anxiety symptoms. On average, participants used the app for 45.83 (SD 111.90) minutes and a total of 5.77 (SD 8.19) days throughout the course of the study. This is not consistent with what participants self-reported, and average app use across the study period was not consistent with what we asked participants to do (use the app for 5 min/d, 5 times/wk). Adherence to web-based and digital interventions is typically lower than that to in-person interventions [72], and future research should implement strategies to promote and ensure participation.

Examining well-being outcomes, objective app use was only significantly associated with an increase in spiritual well-being. Interestingly, improvements in satisfaction with life, religious commitment, and racial or ethnic identity development were not associated with app use. Religious commitment was also high in our sample at baseline as 73% (56/77) always identified with a religion and 26% (20/77) reported that they were new identifiers or reidentifiers, and it may take longer than 4 weeks for a religiously committed sample to observe significant changes in religious commitment. Most participants (57/77, 74%) attended in-person services at least once a week, and almost 25% (18/77, 23%) attended at least once or twice a month, whereas 56% (43/77) participated in a religious group at least once a week and 38% (29/77) participated in a religious group once or twice a month. Religious and spiritual practices outside of an app may be one reason why well-being outcomes significantly improved at the postintervention time point but only spiritual well-being was associated with app use. Future research should consider testing the use of a technology-mediated religious or spiritual practice against samples who attend and do not attend in-person religious services or activities.

In addition, behavior change is complex and may affect individuals in a variety of ways [73]. It is possible that the amount of behavior requested (5 times a week for 4 weeks) was not appropriate for each participant. Although satisfaction with life, religious commitment, and racial or ethnic identity development outcomes were not significantly associated with app use, merely having access to technology-mediated religious or spiritual practices may have contributed to the significant improvements observed. Others have reported that perceived access may be no less valid than actual access and may be a stronger predictor of use than actual access [67,74]. Future research should assess the amount of time spent in digitally mediated religious and spiritual practices that are feasible and elicit changes in well-being outcomes such as satisfaction with life, religious commitment, and racial or ethnic identity development.

### Limitations and Future Research

The results of this study should be interpreted within the context of its limitations. First, this was a feasibility study, and we found initial evidence that the app was perceived as feasible by participants. Although small effect sizes were observed, clinical significance cannot be determined at this time. We encourage future research that uses a more rigorous methodology that relies on longer study periods and random assignment to experimental and control conditions, such as randomized clinical trials. A longer randomized controlled trial would be beneficial for establishing the causal effect of using a faith and prayer app for the purpose of improving mental health and well-being. Randomization and comparison with a control condition will also limit the potential of confounding factors such as possible COVID-19 impact as well as social and economic characteristics. In addition, all outcome measures were self-reported, and the findings should be interpreted based on the potential bias that self-reporting introduces, such as social desirability bias.

Second, a large percentage of participants who initially responded to our survey were determined to be inauthentic responses (eg, bots and duplicate IP addresses). This is a common problem when using the internet and social media to recruit participants. Once we realized the problem, we implemented additional strategies to ensure data quality, such as bot detection and reCAPTCHA and checking responses. Future research should implement these strategies from the beginning of data collection. In addition, it may be helpful to require a face-to-face meeting with potential participants before they enter the study.

Third, our sample predominantly comprised Black or African American individuals who were aged between 26 and 36 years and reported being affiliated with the Christian faith. Our findings may not be generalizable to other REM populations, age groups, or religious or spiritual faiths. For example, religion or spirituality may be experienced differently by older individuals [75], leading to different needs regarding interventions. In addition, as participants self-selected into the study, they may have had expectancy effects regarding the effectiveness of faith-based interventions and may have experienced confirmation bias regarding the effects of using the app. Future work including using purposive sampling with greater racial or ethnic, denominational, and age range diversity would be desirable to test the generalizability of our findings. In addition, information from these studies could be used to help increase the cultural and religious inclusivity of the app.

Fourth, because of the web-based and autonomous nature of the study, we were not able to control how many minutes and days the participants actually engaged with the app. At the onset of their participation, participants were instructed to “engage in the app at least 5x/week and at least 5 minutes/day.” Although our results suggest that greater app use was associated with improved mental health symptoms and increased spiritual well-being, the study results may not have captured the full impact of app use as accurately as a study design in a more controlled setting would have. Future research should strive to establish causal mechanisms of a faith-based app that may elicit improved outcomes and use engagement strategies to ensure adequate and appropriate app use to better capture the relationship between technology-mediated religious and spiritual practice and mental health symptoms and well-being. Future research should also collect subscription rates after the study to increase the validity of the feasibility findings.

### Conclusions

In conclusion, this is the first study to assess the feasibility of a faith and prayer app for mental health (ie, stress, depression, and anxiety) and well-being (ie, religious commitment, spiritual well-being, satisfaction with life, and racial or ethnic identity) in a sample of REM individuals. Our findings suggest that the use of a faith and prayer app (ie, Pray.com) is feasible and may be significantly impactful for the improvement of mental health symptoms and spiritual well-being in REM individuals and their communities, especially Black and African American individuals. Participants also reported some critical feedback about some of the content as well as about the technological interface of the app. These findings should be interpreted based on the preliminary nature of the study and the context of its limitations. This study lays the foundation for future work to be conducted in REM groups to assess the impact of technology-mediated religious or spiritual practice on health and well-being.

## Abbreviations

HADS: Hospital Anxiety and Depression Scale
MEIM: Multigroup Ethnic Identity Measure–Revised
PSS-10: Perceived Stress Scale-10
RCI-10: Religious Commitment Inventory–10
REM: racial and ethnic minority
SWBS: Spiritual Well-Being Scale
SWLS: Satisfaction With Life Scale

## Data Availability

The deidentified data sets generated and analyzed during this study are available from the corresponding author upon reasonable request.
